# Liraglutide for the Treatment of Severe Hypoglycemia Following Total Pancreatectomy and Islet Autotransplantation

**DOI:** 10.1210/jcemcr/luae178

**Published:** 2024-10-24

**Authors:** Christopher Chan, Wayne Hawthorne, Henry Pleass, Deborah Jane Holmes-Walker

**Affiliations:** Department of Diabetes & Endocrinology, Westmead Hospital, Westmead, NSW 2145, Australia; Westmead Clinical School, University of Sydney, Westmead, NSW 2145, Australia; Westmead Clinical School, University of Sydney, Westmead, NSW 2145, Australia; Westmead Institute of Medical Research, Westmead, NSW 2145, Australia; Department of Surgery, Westmead Hospital, Westmead, NSW 2145, Australia; Westmead Clinical School, University of Sydney, Westmead, NSW 2145, Australia; Department of Surgery, Westmead Hospital, Westmead, NSW 2145, Australia; Department of Diabetes & Endocrinology, Westmead Hospital, Westmead, NSW 2145, Australia; Westmead Clinical School, University of Sydney, Westmead, NSW 2145, Australia

**Keywords:** total pancreatectomy, islet cell autotransplantation, hypoglycemia, chronic pancreatitis

## Abstract

Total pancreatectomy and islet autotransplantation (TPIAT) is an effective treatment for chronic and recurrent acute pancreatitis, and it provides a significant potential additional benefit of insulin independence. Spontaneous hypoglycemia in the absence of insulin therapy following TPIAT is a recognized complication, which has been attributed to lack of protective glucagon responses to hypoglycemia, following intrahepatic islet autotransplantation. We describe the use of liraglutide to treat spontaneous hypoglycemia following TPIAT. Continuous glucose monitoring was used to identify timing of hypoglycemia in relation to meals and monitor treatment effect. Liraglutide has been used for management of hypoglycemia following bariatric surgery, but, to our knowledge, this is the first application of its effective use to treat spontaneous severe hypoglycemia following TPIAT.

## Introduction

Chronic pancreatitis is a progressive disorder characterized by persistent inflammation, which includes the destruction of the pancreatic parenchyma and irreversible morphological changes [1]. In this setting, the common etiologies leading to total pancreatectomy and islet autotransplantation (TPIAT) include autoimmune, obstructive, and genetic disorders. It is characterized by severe abdominal pain, caused by inflammation, nerve damage, and ductal obstruction and raised intraductal pressure, and can cause endocrine and exocrine insufficiency, as well as pancreatic ductal adenocarcinoma in the longer term [2]. Treatment has traditionally involved multimodal analgesia, endoscopic interventions such as sphincterotomy and stenting, and eventual surgery, including partial and total pancreatectomy [3].

TPIAT was first performed in the 1970s at Minnesota Children's Hospital and is recommended to be performed earlier in individuals with a known genetic cause for chronic pancreatitis [3, 4]. Spontaneous hypoglycemia following TPIAT has been reported [5], but is incompletely understood, and optimal management is uncertain. We report a case of a patient with chronic pancreatitis secondary to heterozygous *SPINK1*/*CFTR* gene pathogenic variants who was managed with TPIAT achieving insulin independence, but 4 months later developed spontaneous severe hypoglycemia with loss of consciousness, which was refractory to conventional treatment but effectively managed with liraglutide and dietary manipulation.

## Case Presentation

A 16-year-old female patient was referred to our institution for consideration of TPIAT for management of recurrent pancreatitis and chronic pain. Her pain was worse postprandially and limited participation in school and sport. She had a history of recurrent episodes of acute pancreatitis from the age of 13 years; at least 4 of these episodes were associated with raised pancreatic enzymes (lipase 638 U/L, 2227 U/L, 830 U/L, 188 U/L; normal reference range [RR], 8-78 U/L), with subsequent episodes associated with minimal elevation. Genetic testing demonstrated heterozygous *SPINK1 c.101A > G (p.Asn34Ser)* and *CTRC c.247C > G) (p.Arg83Gly)* pathogenic variants.

Her background was significant for Graves disease, celiac disease, pseudohypoparathyroidism (awaiting genetic testing), and pernicious anemia. There is no known association between her genetic pathogenic variants and autoimmune conditions. Medications included regular tapentadol, calcium carbonate, calcitriol, lansoprazole, propylthiouracil, thyroxine, iron tablets, parenteral B12, and the oral contraceptive pill. She took oral pancreatic enzymes on an as-needed basis for fatty meals. She had a family history of type 2 diabetes mellitus but no family history of autoimmune disease. At the time of review, she was in her penultimate year of high school. She otherwise had normal childhood development.

## Diagnostic Assessment

She was seen by the multidisciplinary TPIAT team, including endocrinologist, transplant physician, and transplant surgeon. Her weight was 73.2 kg, height 170 cm, BMI 25.3 kg/m^2^.

She had a normal glycated hemoglobin (HbA1c) of 4.9% (RR, 4.0%-6.0%). Anti-GAD antibodies and anti-ICA antibodies were negative. She had a normal oral glucose tolerance test and good C-peptide response, with mild reactive hypoglycemia to 2.5 mmol/L (45 mg/dL) at the 3-hour mark, somewhat blunting her response to subsequently administered intravenous arginine (Table 1). Oral glucose tolerance testing is routinely performed prior to islet autotransplant to confirm absence of diabetes and to evaluate islet cell reserve with loss of first-phase (elevated 1-hour glucose) and/or second-phase insulin secretion (elevated 2-hour glucose) indicative of loss of islet cell mass and hence likelihood of needing insulin therapy despite islet cell transplantation. This helps to guide the decision as to whether the additional cost of islet cell transplant is worthwhile. Arginine stimulation evaluates an alternate pathway for insulin secretion inducing membrane depolarization at the islet membrane, calcium influx, and subsequent insulin secretion. If the glucose level is concurrently elevated > 15 mmol/L (271 mg/dL) at time of administration of arginine the C-peptide response reflects maximal beta cell secretion [6]. Preoperative C-peptide response to arginine correlates with islet mass available for transplant in pediatric TPIAT recipients [7].

**Table 1.** Preoperative glucose tolerance test and arginine stimulation test

	Time	Glucose	C-peptide	Insulin
Oral glucose (75 grams)	0 minutes	4.1 mmol/L (74 mg/mL)	0.66 nmol/L (1.99 ng/mL)	76 pmol/L (11 mIU/L)
	30 minutes	7.8 mmol/L (140 mg/mL)	2.99 nmol/L (9.03 ng/mL)	625 pmol/L (90 mIU/L)
	60 minutes	8.2 mmol/L (148 mg/mL)	2.53 nmol/L (7.64 ng/mL)	35 pmol/L (5 mIU/L)
	90 minutes	7.1 mmol/L (128 mg/mL)	3.80 nmol/L (11.48 ng/mL)	701 pmol/L (101 mIU/L)
	120 minutes	6.9 mmol/L (124 mg/mL)	4.17 nmol/L (12.59 ng/mL)	771 pmol/L (111 mIU/L)
	150 minutes	6.3 mmol/L (113 gg/mL)	3.31 nmol/L (10.00 ng/mL)	382 pmol/L (55 mIU/L)
IV arginine (30 grams)	180/0 minutes	2.5 mmol/L (45 mg/mL)	1.41 nmol/L (4.26 ng/mL)	69 pmol/L (10 mIU/L)
	16 minutes	3.2 mmol/L (58 mg/mL)	1.06 nmol/L (3.20 ng/mL)	69 pmol/L (10 mIU/L)
	18 minutes	3.2 mmol/L (58 mg/mL)	1.08 nmol/L (3.26 ng/mL)	83 pmol/L (12 mIU/L)
	20 minutes	3.2 mmol/L (58 mg/mL)	1.17 nmol/L (3.53 ng/mL)	76 pmol/L (11 mIU/L)

Reference ranges: Fasting glucose, 3.9-5.4 nmol/L (63-97 mg/mL); C-peptide, 0.26-1.73 nmol/L (0.79-5.23 ng/mL); insulin, <10 mIU/L (<69 pmol/L).

## Treatment

Pre- and postoperatively, dietary education was provided regarding daily carbohydrate distribution and carbohydrate and fat counting in conjunction with education on insulin administration. In October 2021, standard total pancreatectomy was performed with preservation of the splenic vessels and spleen, and standard hepaticojejunostomy and gastroenterostomy without Roux loop, with full perioperative and postoperative heparinization. Additional to the total pancreatectomy, she was given an autoislet transplant. She received a total of 30 mL of islet digest infused into her portal vein with a total of 526 422 islet equivalents [IEQ] or 7192 IEQ/kg of recipient weight. Heparin infusion was discontinued 24 hours postoperatively after doppler confirmed portal vein patency. A blood glucose level (BGL) of 4 to 8 mmol/L (72-144 mg/mL) was targeted using an intravenous insulin-dextrose infusion, with low requirements (0.3-1 unit/h) for the first 4 days. Her diet was gradually increased, and she was changed to multiple daily injections of insulin by postoperative day 7 (total daily insulin [TDI] 9 IU/day). On postoperative day 14, a random nonfasting C-peptide was 0.21 nmol/L (0.63 ng/mL; RR, 0.26-1.73 nmol/L or 0.79-5.23 ng/mL) with a corresponding random plasma glucose of 5.8 mmol/L (105 mg/mL). She was discharged home on insulin aspart and insulin detemir with Freestyle Libre 2™ continuous glucose monitoring (CGM).

## Outcome and Follow-Up

**Figure 1.**
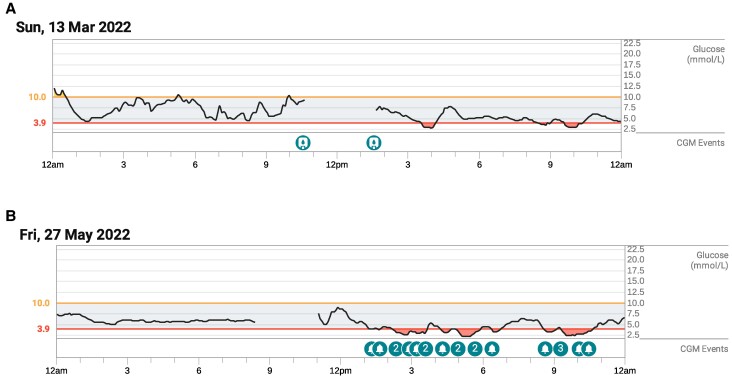
A. CGM trace of postprandial hypoglycemia ∼18 weeks post-TPIAT. B. CGM trace of exercise-induced hypoglycemia ∼24 weeks post-TPIAT. Dexcom™ CGM.

By late December 2021 (7 weeks post-TPIAT), she was tolerating 2 meals per day reliably, with improvement in nausea. Her random C-peptide was 0.67 nmol/L (2.02 ng/mL). She was on insulin detemir 3 units daily, and insulin aspart for larger meals only (> 45 g carbohydrate). She was changed to the DEXCOM G6™ CGM system. By February 2022 (16 weeks post-TPIAT), all analgesia was ceased. Insulin determir was self-ceased, with continued use of insulin aspart alone (TDI 3 IU). A random C-peptide was 2.4 nmol/L (7.25 ng/mL), and HbA1c was 5.4%. At 18 weeks post-TPIAT, she began experiencing episodes of symptomatic postprandial hypoglycemia as low as 2.5 mmol/L on her CGM, several hours following her last dose of insulin aspart (Fig. 1A). The ambulatory glucose profile with sensor wear time of 98% showed < 1% of time very low (BGL < 3.0 mmol/L, 54 mg/dL), 1% low (BGL < 3.9 mmol/L, < 70 mg/dL), 93% in-range (BGL 3.9-10 mmol/L, 70-180 mg/dL), and 6% high (> 10 mmol/L, > 180 mg/dL), with mean glucose 6.5 mmol/L ± 1.6 mmol/L (117 ± 29 mg/dL). All insulin was ceased, and the exercise-induced and postprandial hypoglycemia were initially managed with reinforcement of dietary advice for controlled portions of low glycemic index, complex carbohydrates and avoidance of excessive simple carbohydrates. At 24 weeks post-TPIAT, she began to experience symptomatic hypoglycemia associated initially with aerobic exercise with levels confirmed on fingerstick testing to be 2.5 mmol/L (45 mg/dL) (Fig. 1B). Dietary advice to prevent exercise-induced hypoglycemia was again provided with some improvement. Her calcium/calcitriol and thyroxine replacement required minor uptitration in the initial 12 months following TPIAT, likely related to altered absorption.

**Figure 2.**
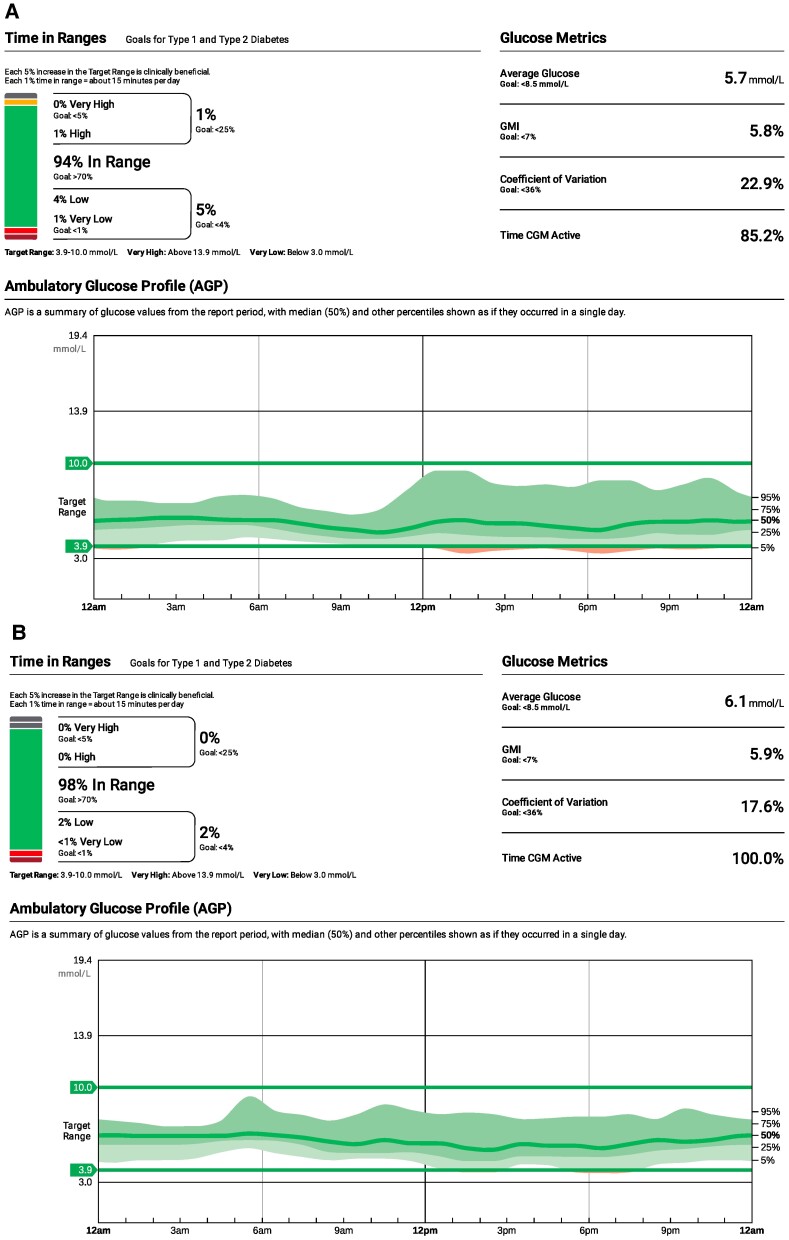
Ambulatory glucose profile comparing hypoglycemia before (A) and after (B) commencement of liraglutide daily therapy. Dexcom^TM^ CGM.

By 18 months post-TPIAT, there was increased frequency of symptomatic postprandial hypoglycemia as low as 2.5 mmol/L (45 mg/dL) on CGM but no episodes of overnight hypoglycemia and no symptomatic fasting hypoglycemia (Fig. 2A). An episode of severe hypoglycemia, with finger stick glucose level of 1.9 mmol/L (34 mg/dL), occurred while she was at a music festival within 2 hours of consumption of 20 g alcohol taken with some complex carbohydrates. She required intravenous glucose with ambulance officers, and she was monitored in the hospital overnight. She was not wearing CGM at the time of the episode. Morning cortisol was normal at 557 nmol/L (20.2 ug/dL; RR, 70-650 nmol/L [2.5-23.6 ug/dL]), thyroid function tests were normal (thyrotropin [TSH] 2.44 mIU/L [RR, 0.40-3.50 mIU/L]; free thyroxine [T4] 14.6 pmol/L [RR, 9.0-19.0 pmol/L], free triiodothyronine [T3] 3.8 pmol/L [RR, 2.6-6.0 pmol/L]) on antithyroid medication, and calcium levels were normal.

She was commenced on acarbose 25 mg with meals, and received further dietary advice to increase complex, low glycemic index carbohydrate. Although symptomatic hypoglycemia frequency and severity was reduced with acarbose, gastrointestinal side effects limited its use. She was changed to liraglutide 0.6 mg daily subcutaneously (20 months post-TPIAT), which was uptitrated to 1.2 mg daily over the following 2 weeks. She experienced mild gastrointestinal side effects with liraglutide initially. There was symptomatic improvement in the frequency of the hypoglycemic episodes both with exercise and postprandially, confirmed on the ambulatory glucose profile (Fig. 2B), and marked improvement in her symptoms and functioning. She had no further episodes of severe hypoglycemia (grade 3).

## Discussion

To our knowledge, this is the first documented use of a glucagon-like peptide (GLP-1) receptor agonist (GLP-1 RA) used to treat hypoglycemia following TPIAT. Increasing numbers of patients are being treated with TPIAT because it offers insulin independence in approximately 50% at 2 years [8], and the prevention of unstable glycemic levels in all who undergo the procedure. Preoperative dynamic testing is performed routinely at most centers, but the optimal metabolic pre-TPIAT assessment (glucose tolerance vs mixed meal testing, etc) is uncertain; however, abnormal glycemia is a consistent predictor of insulin-dependence postoperatively [9-11]. Those receiving a higher islet cell mass are more likely to achieve insulin independence and usually associated with earlier intervention with minimal surgical intervention prior to TPIAT [12].

In a cohort of 40 TPIAT recipients over 7 years from the Cleveland Clinic, 6 of 12 recipients developed symptomatic and severe hypoglycemia between 1 and 12 months following insulin independence [5]. Bellin et al demonstrated in a hyperinsulinemic hypoglycemic clamp study that individuals with autoislet transplants in the liver have defective glucagon response to hypoglycemia compared to healthy controls and compared to those with autoislet transplants in both the liver and other extrahepatic sites [13]. This is postulated to result from the increased intrahepatic glucose flux that results following gluconeogenesis and glycogenolysis in response to hypoglycemia, blocking the stimulus for α-cells lodged within hepatic sinusoids to release glucagon despite systemic hypoglycemia [5, 13, 14]. Deficient central nervous system innervation of intrahepatic autoislets, usually a stimulus for native α-cell production of glucagon, is another possible contributor but thought to be less likely as transplanted pancreas in the pelvis have been shown to have normal glucagon responses to hypoglycemia [13, 15]. The application of glucagon microdosing with multiple daily injections has recently been reported for treatment of spontaneous hypoglycemia following TPIAT [16].

Altered delivery of ingested foods due to small bowel resection has been postulated to be contributory to postprandial hypoglycemia following TPIAT, similar to that reported in postbariatric hypoglycemia (PBH). It is thought to be mediated by several factors, including accelerated nutrient delivery to the small intestine, an exaggerated incretin response, and resultant postprandial hyperinsulinemia [17]. Impaired counterregulatory responses are thought to contribute, including a reduction in glucagon response to hypoglycemia [18], similar to what has been demonstrated in mixed meal tests in patients following TPIAT [19]. Unlike the patients in Lin et al's cohort [5], our patient's hypoglycemia occurred predominantly postprandially. Thus, we chose agents that have previously been used successfully to manage PBH; acarbose has been shown to be effective in small studies for PBH [20, 21] and liraglutide in case studies [22]. In studies of individuals postbariatric surgery, those who experienced PBH have been shown to have correspondingly higher levels of postmeal GLP-1 levels compared to those without PBH [23]. GLP-1 RAs slow the delivery of ingested nutrients to the small bowel, thus reducing the postprandial GLP-1 rise and preventing excessive activation of the GLP-1 receptor and subsequent insulin release [24]. A recent paper has also proposed that GLP-1 RAs act differently from native GLP-1 due to the constant stimulation of the receptor as compared with intermittent stimulation postprandially [25]. We postulate that the combination of a PBH-like syndrome and reduction in protective mechanisms against hypoglycemia, including deficient glucagon responses to hypoglycemia, likely explain the effectiveness of liraglutide in our patient.

The relationship of reactive hypoglycemia on the preoperative glucose tolerance test and the hypoglycemia following TPIAT are likely unrelated. There was no history of postprandial symptoms of hypoglycemia prior to surgery. Additionally, our case only explored the effects of a daily administered GLP-1 RA. Newer GLP-1 RAs administered weekly or orally may elicit different clinical responses.

In conclusion, we report the benefit of daily subcutaneous liraglutide to treat spontaneous severe hypoglycemia despite insulin independence following TPIAT. Its success highlights mechanistic similarities between PBH and hypoglycemia following TPIAT.

## Learning Points

- TPIAT with intrahepatic infusion of islet cells is associated with a risk of spontaneous hypoglycemia in those who achieve insulin independence. This is due to a combination of reduced glucagon response and altered nutrient delivery to the small intestine, which likely alters GLP-1 secretion similar to that demonstrated following bariatric surgical procedures.
- Agents such as GLP-1 receptor agonists used in the management of postbariatric hypoglycemia are efficacious in TPIAT-associated hypoglycemia, particularly if the hypoglycemia is postprandial.
- Continuous glucose monitoring is useful to assess timing of spontaneous hypoglycemia following TPIAT and response to treatment.

## Contributors

All authors made individual contributions to writing of the paper. D.J.H.W., H.P., and W.H. were involved in the diagnosis and the initial and ongoing management of the patient. C.C. was involved in the therapeutic management of the patient. All authors reviewed and approved the final draft.

## Data Availability

Data sharing is not applicable to this article as no datasets were generated or analyzed during the current study.

## Abbreviations

BGL: blood glucose level
CGM: continuous glucose monitoring
GLP-1: glucagon-like peptide 1
GLP-1 RA: glucagon-like peptide 1 receptor agonist
HbA1c: glycated hemoglobin
PBH: postbariatric hypoglycemia
RR: reference range
TDI: total daily insulin
TPIAT: total pancreatectomy and islet autotransplantation
